# Association between nontraditional lipid profiles and the severity of obstructive sleep apnea: A retrospective study

**DOI:** 10.1002/jcla.24499

**Published:** 2022-05-16

**Authors:** Yifei Fang, Jiao Su, Chunling Zhao, Yang Meng, Beilei Wei, Binglu Zhang, Yuyang Huang, Liping Dai, Songyun Ouyang

**Affiliations:** ^1^ Department of Respiratory and Sleep Medicine The First Affiliated Hospital of Zhengzhou University Zhengzhou Henan China; ^2^ Henan Institute of Medical and Pharmaceutical Sciences Zhengzhou University Zhengzhou Henan China

**Keywords:** cardiovascular diseases, dyslipidemia, nontraditional lipid profiles, obstructive sleep apnea, severe OSA

## Abstract

**Background:**

Due to the significant role of dyslipidemia, cardiovascular diseases (CVDs) are very common in obstructive sleep apnea (OSA). Nontraditional lipid indices are considered to be a better predictive index for cardiovascular risk. Nevertheless, the association between nontraditional lipid profiles and the severity of OSA is not clear.

**Methods:**

A retrospective study was proceeded on 635 patients. Subjects were diagnosed with OSA through polysomnography (PSG). The association between severe OSA and nontraditional lipid profiles [triglyceride (TG)/high‐density lipoprotein cholesterol (HDL‐C) ratio, total cholesterol (TC)/HDL‐C ratio, low‐density lipoprotein cholesterol (LDL‐C)/HDL‐C ratio, non‐high‐density lipoprotein cholesterol (non‐HDL‐C), atherogenic index (AI), and lipoprotein combine index (LCI)] was examined by utilizing the restricted cubic spline and multivariate logistic regression analysis.

**Results:**

All nontraditional lipid indices had positive relationships with the severity of OSA. By multivariable adjustment, the per SD increment of the TG/HDL‐C, TC/ HDL‐C, LDL‐C/HDL‐C, non‐HDL‐C, AI, and LCI were significantly associated with 88%, 50%, 42%, 40%, 50%, and 125% higher risk for severe OSA respectively. Compared with the lowest tertiles, the adjusted ORs (95% CI) were 2.42 (1.57–3.75), 2.39 (1.53–3.73), 2.35 (1.52–3.64), 1.86 (1.21–2.86), 2.39 (1.53–3.73), and 2.23 (1.43–3.48) for the top tertiles of TG/HDL‐C, TC/ HDL‐C, LDL‐C/HDL‐C, non‐HDL‐C, AI, and LCI respectively.

**Conclusion:**

All nontraditional lipid indices had positive relationship with the severity of OSA. In addition, TG/HDL‐C, TC/HDL‐C, and AI had better performance than the other nontraditional lipid indices for predicting severe OSA. These findings could help to determine the risk of cardiovascular diseases and improve the dyslipidemia management of OSA patients.

## Graphical abstract

This study is different from previous studies. Firstly, as a retrospective cohort study, the sample size is larger than in other studies. Secondly, previous studies only investigated 1 or 2 nontraditional lipid indexes, while our research studied 6 nontraditional lipid indexes. Last but not least, the restricted cubic spline is employed in the research to confirm the linearly positive association between nontraditional lipid profiles and the risk of severe OSA.

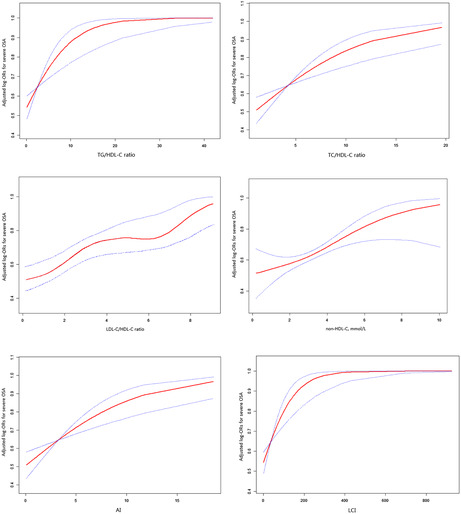

## 1 INTRODUCTION

As a widespread respiratory disorder, obstructive sleep apnea (OSA) is characterized by intermittent, complete, and partial airway collapse of the upper airway during the patient's sleep. It could lead to frequent episodes of apnea and hypopnea.^1^ OSA is closely associated with other diseases such as cardiovascular diseases (CVDs), metabolic syndrome, insulin resistance, and type 2 diabetes mellitus.^2^ Some mechanisms between OSA and cardiovascular outcomes have been widely studied. For example, sympathetic activation, chronic intermittent hypoxia, oxidative stress, and systemic inflammation are associated with metabolic dysfunction, which may explain the lipid metabolism abnormalities in OSA.^3^, ^4^ Dyslipidemia is a well‐known risk factor for the progression and development of CVDs. As a vital component of metabolic syndrome, it includes hypertension, heart failure, and coronary artery disease.^5^, ^6^ Thus, grasping the relationship between lipid metabolism and OSA is of great significance to guide the management and treatment of OSA.

For the past few years, more and more researchers have been focusing on the possible correlation between lipid metabolism disorders and OSA, especially in traditional lipids. There is a correlation between OSA and the increase in total cholesterol (TC), triglycerides (TG), and low‐density lipoprotein cholesterol (LDL‐C). In addition, the decrease in high‐density lipoprotein cholesterol (HDL‐C) is also related to OSA.^7^, ^8^, ^9^ Compared with traditional lipid parameters, the nontraditional lipid indexes, including TG/HDL‐C, TC/HDL‐C, LDL‐C/HDL‐C, non‐HDL‐C, non‐HDL‐C/HDL‐C (atherogenic index, AI), and TC*TG*LDL‐C/HDL‐C (lipoprotein combine index, LCI), are believed to be better predictors for CVD.^10^, ^11^ Yet even the nontraditional lipid profiles serve as sensitive indexes for CVDs risk, the complex interplay in OSA is not fully understood. Therefore, the aim of our research is to explore the relationship of OSA severity with nontraditional lipid profiles, which could help to determine the risk of cardiovascular diseases and improve the dyslipidemia management in OSA patients.

Based on the following aspects, this study is different from previous studies. Firstly, as a retrospective cohort study, the sample size is larger than in other studies. ^12^ Secondly, previous studies only investigated 1 or 2 nontraditional lipid indexes,^13^, ^14^ while our research studied 6 nontraditional lipid indexes. Last but not least, the restricted cubic spline is employed in the research to confirm the linearly positive association between nontraditional lipid profiles and the risk of severe OSA.

## 2 METHODS

### 2.1 Study population

**FIGURE 1:**
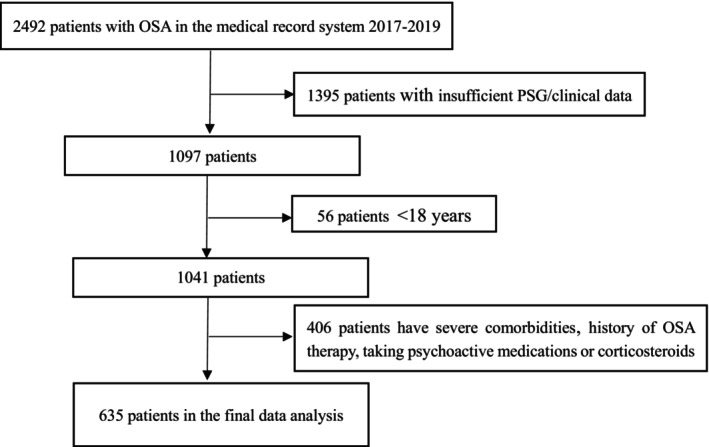
Flow chart of the study

A retrospective research of 2492 subjects diagnosed with OSA via polysomnography (PSG) test was carried out between January 2017 and December 2019. Data were collected from the medical record system in a retrospective manner, including sex, age, height, weight, body mass index (BMI), past history (hypertension, diabetes mellitus, coronary artery disease, and cerebral vascular disease), personal history (smoking and alcohol drinking), sleep parameters, fasting plasma lipids, and patients' blood pressure (BP). The body mass index (BMI) was calculated by dividing the weight by the height squared. The patients' BP was measured in the evening before PSG and in the morning after PSG. Six hundred thirty‐five subjects remained after the following exclusion criteria: (1) individuals under the age of 18; (2) individuals with insufficient polysomnographic/clinical data; (3) individuals with history of OSA therapy; (4) individuals with corticosteroids or taking psychoactive medications; and (5) individuals with severe heart failure, liver or kidney diseases, or malignant tumor (see Figure 1).

### 2.2 Polysomnography

Overnight PSG (SOMNOmedics GmbH, Randersacker, Germany) was performed at the sleep center in the First Affiliated Hospital of Zhengzhou University. Sleep and associated events were scored according to the guidelines of the American Academy of Sleep Medicine (AASM).^15^ Apnea was defined as a ≥90% drop in flow for at least 10s. Hypopnea was defined as a 30% or greater drop in breathing amplitude for more than 10s and accompanied by greater than 3% decrease in oxyhemoglobin saturation or an arousal. Apnea–hypopnea index (AHI) was employed to measure the average number of apnea and hypopnea per hour. Mild, moderate, and severe OSA were defined as AHI of 5–15, 16–30, and >30 events/hour respectively. Mild and moderate OSA were collectively called non‐severe OSA. Oxygen desaturation index (ODI) was defined as at least 3% decrease in saturation per hour. The mean value and minimum value of SaO_2_ were also recorded in PSG.

### 2.3 Biochemical parameters

Fasting plasma lipids included total cholesterol (TC), triglycerides (TG), high‐density lipoprotein cholesterol (HDL‐C), and low‐density lipoprotein cholesterol (LDL‐C). After more than 12 h of overnight fasting, all venous blood samples of the study participants were collected. TC, TG, HDL‐C, and LDL‐C were measured by enzymatic methods directly. All the measurements were carried out by employing an automatic clinical chemistry analyzer (Beckman Coulter AU5800, USA). According to the separated lipid parameters, the following clinical indicators were calculated: TC/HDL‐C ratio, TG/HDL‐C ratio, LDL‐C/HDL‐C ratio, AI, LCI, and non‐HDL‐C. Non‐HDL‐C was calculated as TC minus HDL‐C; AI was calculated as non‐HDL‐C/HDL‐C; and LCI was calculated as TC*TG*LDL‐C/HDL‐C.

### 2.4 Statistical analyses

The number (%) and the mean ± SD or the median (25th percentile and 75th percentile) were utilized to describe continuous data and categorical data respectively. By employing the one‐way analysis of variance (ANOVA) and chi‐squared test for categorical data, differences between mild, moderate, and severe OSA in data characteristics were compared. The univariate linear regression was utilized to evaluate the associations between sleep parameters and nontraditional lipid indices. By presenting the 95% confidence interval (CI) and the odds ratio (OR) with adjustment for covariates (age, sex, BMI, smoking, alcohol drinking, hypertension, diabetes mellitus, coronary artery disease, and cerebral vascular disease), the association between nontraditional lipid indices and OSA was assessed via logistic regression analyses. In the assessing of nontraditional lipid profiles, we established models for both continuous data per SD increment and categorical data by utilizing tertiles with the lowest tertiles (T1) as the reference group. Restricted cubic spline (smooth fitting curve) was performed to visually evaluate the association with nontraditional lipid profiles of the severity of OSA. The tests for linear trend were performed by entering the tertiles of each category of nontraditional lipid profile as continuous data in the models. Interaction and subgroup analyses were conducted according to sex, age (<65 and ≥65 years), BMI (<24 kg/m^2^ and ≥24 kg/m^2^), smoking, alcohol drinking, hypertension, diabetes mellitus, coronary artery disease, and cerebral vascular disease. All the data analyses were performed by employing R version 3.6.1 (www.R‐project.org) and EmpowerStates (www. empowerstats.com). A 2‐sided *p* < 0.05 was considered to be statistically significant.

## 3 RESULTS

### 3.1 Baseline characteristics

The clinical and biochemical data are shown in Table 1. Based on AHI, 88 subjects were assigned to mild group; 137 subjects were assigned to moderate group; and 410 subjects were assigned to severe group. Our research showed significant differences in sex, BMI, smoking, alcohol drinking, hypertension, cerebral vascular disease, TC, TG, LDL‐C, HDL‐C, TG/HDL‐C, TC/HDL‐C, LDL‐C/HDL‐C, non‐HDL‐C, AI, LCI, AHI, ODI, mean SaO_2_, minimum SaO_2,_ and morning BP (*p* < 0.05). No significant differences were found in terms of age, diabetes mellitus, coronary artery disease, and evening BP.

**TABLE 1:** Characteristics of study participants

Variables	Mild OSA (*n* = 88)	Moderate OSA (*n* = 137)	Severe OSA (*n* = 410)	*p* value
Age, year	47.59 ± 13.62	48.74 ± 12.11	47.70 ± 12.72	0.690
Male, *n* (%)	55 (62.50%)	109 (79.56%)	340 (82.93%)	<0.001^***^
BMI, kg/m^2^	27.43 ± 4.04	28.49 ± 5.31	30.57 ± 5.99	<0.001^***^
Smoking, *n* (%)	23 (26.14%)	57 (41.61%)	180 (43.90%)	0.009^**^
Alcohol drinking, *n* (%)	22 (25.00%)	52 (37.96%)	164 (40.00%)	0.031^*^
Hypertension, *n* (%)	34 (38.64%)	74 (54.01%)	230 (56.10%)	0.012^*^
Diabetes mellitus, *n* (%)	9 (10.23%)	30 (21.90%)	85 (20.73%)	0.058
Coronary artery disease, *n* (%)	12 (13.64%)	29 (21.17%)	89 (21.71%)	0.229
Cerebral vascular disease, *n* (%)	9 (10.23%)	21 (15.33%)	28 (6.83%)	0.011^*^
TC, mmol/L	4.16 ± 0.89	4.44 ± 1.01	4.61 ± 1.25	0.003^**^
TG, mmol/L	1.58 (0.98–2.12)	1.62 (1.08–2.47)	2.01 (1.34–2.94)	<0.001^***^
LDL‐C, mmol/L	2.22 ± 0.94	2.32 ± 1.04	2.53 ± 1.13	0.016^*^
HDL‐C, mmol/L	1.25 (0.97–1.71)	1.11 (0.92–1.60)	0.99 (0.82–1.34)	<0.001^***^
TG/HDL‐C	0.96 (0.61–2.04)	1.38 (0.75–2.30)	1.83 (0.97–3.28)	<0.001^***^
TC/HDL‐C	3.23 (2.01–4.16)	3.76 (2.25–5.04)	4.22 (2.99–5.60)	<0.001^***^
LDL‐C /HDL‐C	1.91 (0.84–2.71)	2.19 (0.96–3.15)	2.64 (1.64–3.61)	<0.001^***^
Non‐HDL‐C, mmol/L	2.71 ± 1.08	2.97 ± 1.16	3.32 ± 1.43	<0.001^***^
AI	2.23 (1.01–3.16)	2.76 (1.25–4.04)	3.22 (1.99–4.60)	<0.001^***^
LCI	10.62 (3.52–22.58)	14.02 (4.14–27.99)	20.88 (7.65–47.28)	<0.001^***^
AHI	9.84 ± 2.79	20.59 ± 3.78	53.28 ± 18.11	<0.001^***^
ODI	15.60 (9.80–34.98)	28.00 (17.10–45.70)	48.15 (35.42–64.10)	<0.001^***^
Mean SaO_2_, %	93.92 ± 3.61	93.01 ± 4.21	89.96 ± 5.95	<0.001^***^
Minimum SaO_2_, %	82.65 ± 10.76	78.27 ± 12.19	68.47 ± 11.76	<0.001^***^
Evening SBP, mm Hg	137.99 ± 19.35	140.23 ± 20.14	142.87 ± 19.15	0.065
Evening DBP, mm Hg	86.59 ± 14.85	86.26 ± 14.41	88.88 ± 12.91	0.085
Morning SBP, mm Hg	132.65 ± 17.23	136.09 ± 16.98	140.84 ± 17.34	<0.001^***^
Morning DBP, mm Hg	85.64 ± 14.51	86.42 ± 14.53	90.50 ± 13.39	<0.001^***^

Abbreviations: AHI, apnea–hypopnea index; AI, atherogenic index; BMI, body mass index; DBP, diastolic blood pressure; HDL‐C, high‐density lipoprotein cholesterol; LCI, lipoprotein combine index; LDL‐C /HDL‐C, low‐density lipoprotein cholesterol to high‐density lipoprotein cholesterol; LDL‐C, low‐density lipoprotein cholesterol; ODI, oxygen desaturation index; SaO_2_, oxygen saturation; SBP, systolic blood pressure; TC, total cholesterol; TC/HDL‐C, total cholesterol to high‐density lipoprotein cholesterol; TG, triglycerides; TG/HDL‐C, triglycerides to high‐density lipoprotein cholesterol ratio.

^*^ *p* < 0.05

^**^ *p* < 0.01

^***^ *p* < 0.001.

### 3.2 Univariate analysis

The results of univariate analysis are shown in Table 2. It could be found that TG/HDL‐C, TC/HDL‐C, LDL‐C /HDL‐C, non‐HDL‐C, AI, and LCI were correlated with higher AHI and ODI.

**TABLE 2:** Results of univariate analysis

	AHI		ODI		Minimum SaO_2_	
	Effect size (*β*)	*p* value	Effect size (*β*)	*p* value	Effect size (*β*)	*p* value
TG/HDL‐C	1.50 (0.97–2.02)	<0.001^***^	1.44 (0.89–1.99)	<0.001^***^	−0.63 (−0.93 to −0.33)	<0.001^***^
TC/HDL‐C	2.28 (1.49–3.07)	<0.001^***^	1.92 (1.08–2.75)	<0.001^***^	−1.12 (−1.57 to −0.68)	<0.001^***^
LDL‐C/HDL‐C	3.05 (1.92–4.18)	<0.001^***^	2.40 (1.21–3.58)	<0.001^***^	−1.57 (−2.20 to −0.93)	<0.001^***^
Non‐HDL‐C	3.49 (2.17–4.80)	<0.001^***^	2.59 (1.21–3.97)	<0.001^***^	−1.63 (−2.37 to −0.89)	<0.001^***^
AI	2.28 (1.49–3.07)	<0.001^***^	1.92 (1.08–2.75)	<0.001^***^	−1.12 (−1.57 to −0.68)	<0.001^***^
LCI	0.07 (0.04–0.10)	<0.001^***^	0.06 (0.03–0.09)	<0.001^***^	−0.03 (−0.05 to −0.02)	<0.001^***^

Abbreviations: AHI, apnea–hypopnea index; AI, atherogenic index; LCI, lipoprotein combine index; LDL‐C /HDL‐C, low‐density lipoprotein cholesterol to high‐density lipoprotein cholesterol; ODI, oxygen desaturation index; SaO_2_, oxygen saturation; TC/HDL‐C, total cholesterol to high‐density lipoprotein cholesterol; TG/HDL‐C, triglycerides to high‐density lipoprotein cholesterol ratio.

^***^ *p* < 0.001.

### 3.3 Nontraditional lipid profiles and OSA

**FIGURE 2:**
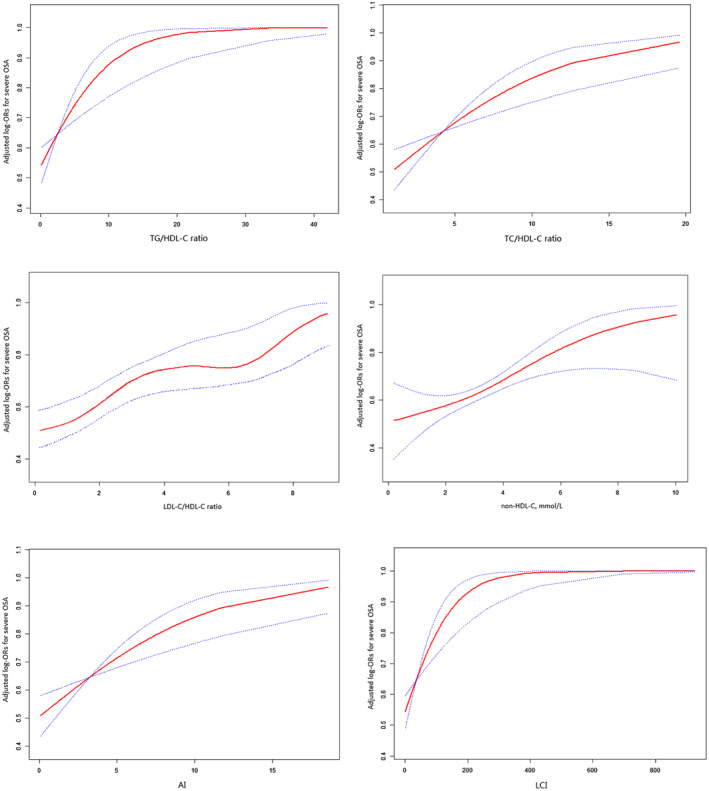
The association between the TG/HDL‐C, TC/HDL‐C, LDL‐C/HDL‐C, non‐HDL‐C, AI, and LCI levels and the risk of severe OSA. The solid line and dashed line represent the estimated values and their corresponding 95% confidence interval, respectively. The adjustment factors included age, sex, BMI, smoking, alcohol drinking, hypertension, diabetes mellitus, coronary artery disease, and cerebral vascular disease

The crude and adjusted models are shown in Table 3. In crude model, all nontraditional lipid profiles were significantly and positively associated with severe OSA. In model 1, the per SD increment of TG/HDL‐C ratio, TC/ HDL‐C ratio, LDL‐C/HDL‐C ratio, non‐HDL‐C, AI, and LCI were significantly associated with a 88%, 50%, 42%, 40%, 50%, and 125% higher risk for severe OSA respectively. Compared with the lowest tertiles, the adjusted ORs (95% CI) were 2.42 (1.57–3.75), 2.39 (1.53–3.73), 2.35 (1.52–3.64), 1.86 (1.21–2.86), 2.39 (1.53–3.73), and 2.23 (1.43–3.48) for the top tertiles of the TG/HDL‐C ratio, TC/HDL‐C ratio, LDL‐C/HDL‐C ratio, non‐HDL‐C, AI, and LCI, respectively, after assessing the nontraditional lipid profiles as tertiles. The result of model 2 was similar to model 1. The association between nontraditional lipid profiles and severe OSA was likely to be linear (all *p* for trend<0.05). Further analyses by restricted cubic spline confirmed the linearly positive association between nontraditional lipid profiles and the risk of severe OSA (see Figure 2).

**TABLE 3:** Odds ratio of severe OSA according to continuous or tertiles of nontraditional lipid profiles

Variables	Crude		Model 1		Model 2	
	OR (95% CI)	*p* value	OR (95% CI)	*p* value	OR (95% CI)	*p* value
TG/HDL‐C (Per 1 SD increase)	2.07 (1.49–2.86)	<0.001^***^	1.88 (1.34–2.62)	<0.001^***^	1.84 (1.31–2.57)	<0.001^***^
Tertiles of TG/HDL‐C						
T1 (<1.03)	1.00 (reference)		1.00 (reference)		1.00 (reference)	
T2 (1.03–2.25)	1.99 (1.34–2.95)	<0.001^***^	1.73 (1.15–2.62)	<0.01^**^	1.77 (1.16–2.69)	<0.01^**^
T 3(≥2.25)	2.76 (1.84–4.17)	<0.001^***^	2.42 (1.57–3.75)	<0.001^***^	2.39 (1.53–3.72)	<0.001^***^
*p* for trend		<0.001^***^		<0.001^***^		<0.001^***^
TC/HDL‐C (Per 1 SD increase)	1.58 (1.30–1.91)	<0.001^***^	1.50 (1.23–1.84)	<0.001^***^	1.49 (1.21–1.82)	<0.001^***^
Tertiles of TC/HDL‐C						
T1 (<3.23)	1.00 (reference)		1.00 (reference)		1.00 (reference)	
T2 (3.23–4.77)	1.38 (0.94–2.04)	0.102	1.43 (0.95–2.15)	0.086	1.42 (0.94–2.15)	0.095
T3 (≥4.77)	2.68 (1.77–4.07)	<0.001^***^	2.39 (1.53–3.73)	<0.001^***^	2.34 (1.49–3.66)	<0.001^***^
*p* for trend		<0.001^***^		<0.001^***^		<0.001^***^
LDL‐C/HDL‐C (Per 1 SD increase)	1.47 (1.23–1.75)	<0.001^***^	1.42 (1.18–1.72)	<0.001^***^	1.42 (1.18–1.72)	<0.001^***^
Tertiles of LDL‐C /HDL‐C						
T1 (<1.85)	1.00 (reference)		1.00 (reference)		1.00 (reference)	
T2 (1.85–2.97)	1.63 (1.10–2.40)	0.015	1.73 (1.15–2.61)	0.009^**^	1.73 (1.14–2.63)	0.010
T3 (≥2.97)	2.52 (1.67–3.79)	<0.001^***^	2.35 (1.52–3.64)	<0.001^***^	2.32 (1.49–3.62)	<0.001^***^
*p* for trend		<0.001^***^		<0.001^***^		<0.001^***^
Non‐HDL‐C(Per 1 SD increase)	1.44 (1.20–1.71)	<0.001^***^	1.40 (1.16–1.68)	<0.001^***^	1.41 (1.16–1.70)	<0.001^***^
Tertiles of non‐HDL‐C						
T1 (<2.49)	1.00 (reference)		1.00 (reference)		1.00 (reference)	
T2 (2.49–3.64)	1.14 (0.77–1.68)	0.524	1.13 (0.75–1.71)	0.550	1.14 (0.74–1.73)	0.556
T3 (≥3.64)	1.97 (1.31–2.96)	0.001^**^	1.86 (1.21–2.86)	0.005^**^	1.92 (1.23–2.98)	0.004^**^
*p* for trend		0.001^**^		0.005^**^		0.004^**^
AI (Per 1 SD increase)	1.58 (1.30–1.91)	<0.001^***^	1.50 (1.23–1.84)	<0.001^***^	1.49 (1.21–1.82)	<0.001^***^
Tertiles of AI						
T1 (<2.23)	1.00 (reference)		1.00 (reference)		1.00 (reference)	
T2 (2.23–3.77)	1.38 (0.94–2.04)	0.102	1.43 (0.95–2.15)	0.086	1.42 (0.94–2.15)	0.095
T3 (≥3.77)	2.68 (1.77–4.07)	<0.001^***^	2.39 (1.53–3.73)	<0.001^***^	2.34 (1.49–3.66)	<0.001^***^
*p* for trend		<0.001^***^		<0.001^***^		<0.001^***^
LCI (Per 1 SD increase)	2.40 (1.64–3.53)	<0.001^***^	2.25 (1.52–3.34)	<0.001^***^	2.22 (1.49–3.30)	<0.001^***^
Tertiles of LCI						
T1 (<8.92)	1.00 (reference)		1.00 (reference)		1.00 (reference)	
T2 (8.92–29.48)	1.07 (0.73–1.58)	0.724	0.98 (0.65–1.47)	0.926	0.99 (0.65–1.49)	0.944
T3 (≥29.48)	2.45 (1.61–3.73)	<0.001^***^	2.23 (1.43–3.48)	<0.001^***^	2.20 (1.40–3.46)	<0.001^***^
*p* for trend		<0.001^***^		<0.001^***^		<0.001^***^

Abbreviations: AI, atherogenic index; LCI, lipoprotein combine index; LDL‐C /HDL‐C, low‐density lipoprotein cholesterol to high‐density lipoprotein cholesterol; TC/HDL‐C, total cholesterol to high‐density lipoprotein cholesterol; TG/HDL‐C, triglycerides to high‐density lipoprotein cholesterol ratio.

Model 1 was adjusted for age, sex, BMI, smoking, alcohol drinking;

Model 2 was adjusted for age, sex, BMI, smoking, alcohol drinking, hypertension, diabetes mellitus, coronary artery disease, cerebral vascular disease.

^**^ *p* < 0.01

^***^ *p* < 0.001.

### 3.4 Subgroup analyses

As shown in Table 4, the test for interactions was significant for sex in TG/HDL‐C ratio (*p* for interaction = 0.0255), while the test for interactions was not significant for age, BMI, smoking, alcohol drinking, hypertension, diabetes mellitus, coronary artery disease, and cerebral vascular disease (all *p*‐interaction >0.05). TG/HDL‐C was a risk factor for severe OSA in female (OR = 5.50, 95% CI [1.74–17.41]). None of the stratified variables, including age, sex, BMI, smoking, alcohol drinking, hypertension, diabetes mellitus, coronary artery disease, and cerebral vascular disease significantly modified the association between nontraditional lipid profiles (TC/HDL‐C, LDL‐C/HDL‐C, non‐HDL‐C, AI, and LCI) with the risk of severe OSA (all *p*‐interaction >0.05) (see Additional file 1: Tables S1–S5).

**TABLE 4:** Association between TG/HDL‐C ratio (per SD increment) and the risk of severe OSA in various subgroups

Subgroups	N	OR (95% CI)	*p* value	*p* for interaction
Sex				0.026^*^
Male	504	1.58 (1.13–2.19)	0.007^**^	
Female	131	5.50 (1.74–17.41)	0.004^**^	
Age (year)				0.200
<65	571	1.96 (1.36–2.83)	< 0.001^***^	
≥65	64	0.97 (0.38–2.48)	0.950	
BMI (kg/m^2^)				0.360
<24	61	1.12 (0.35–3.62)	0.847	
≥24	574	2.02 (1.40–2.90)	< 0.001^***^	
Smoking				0.290
No	375	2.19 (1.38–3.47)	<0.001^***^	
Yes	260	1.50 (0.90–2.50)	0.118	
Alcohol drinking				0.201
No	397	2.31 (1.43–3.74)	<0.001^***^	
Yes	238	1.47 (0.89–2.41)	0.129	
Hypertension				0.840
No	297	1.80 (1.08–2.98)	0.024^*^	
Yes	338	1.93 (1.22–3.04)	0.005^**^	
Diabetes mellitus				0.565
No	511	1.96 (1.33–2.90)	<0.001^***^	
Yes	124	1.57 (0.83–2.96)	0.168	
Coronary artery disease				0.163
No	505	2.06 (1.38–3.08)	<0.001^***^	
Yes	130	1.21 (0.67–2.18)	0.528	
Cerebral vascular disease				0.053
No	577	2.03 (1.40–2.96)	<0.001^***^	
Yes	58	0.72 (0.26–1.97)	0.527	

Abbreviation: BMI, body mass index.

Adjusted for: age, sex, BMI, smoking, alcohol drinking, hypertension, diabetes mellitus, coronary artery disease, cerebral vascular disease. In each case, the model is not adjusted for the stratification variable.

^*^ *p* < 0.05

^**^ *p* < 0.01

^***^ *p* < 0.001.

## 4 DISCUSSION

This study explored the relationship of OSA severity with nontraditional lipid profiles. Our results revealed that the nontraditional lipid profiles were all positively related to the risk of severe OSA. The restricted cubic spline denoted that the relationship of the six nontraditional lipid indices with severe OSA was linear. Moreover, the findings suggested that TG/HDL‐C ratio, TC/ HDL‐C ratio, and AI could be utilized as better predictors of severe OSA risk than other nontraditional lipid indices after adjusting for confounders. Besides, the association between TG/HDL‐C ratio and severe OSA was modified by sex. As far as we know, this is the first study that showed the independent association of OSA severity with nontraditional lipid profiles.

The TG/HDL‐C ratio or its logarithm has been discussed only in few studies in OSA. A Japanese research found that OSA was associated with increased atherogenic index of plasma (AIP), but only in males with normal weight.^13^ TG/HDL‐C ratio was significantly associated with AHI in severe group after adjusting for confounding variables in a cross‐sectional study of 246 male bus drivers.^12^ The above‐mentioned studies investigated men exclusively, while the relationship between OSA and TG/HDL‐C ratio also depends on gender.^16^ Compared with controls, TG/HDL‐C ratio increased in mild OSA group.^17^ Nevertheless, compared with the previous studies, our cohort had a wider representation of age, gender, comorbidities, and the severity of OSA. Our study showed that TG/HDL‐C ratio was related to the risk of severe OSA and was more pronounced in female patients with severe OSA. Although TG/HDL‐C ratio could predict for the risk of severe OSA in both men and women, it was more significant for women. It is possible that women who lose the protection from estrogen have greater serious dyslipidemia in severe OSA.^18^

Researchers found an independent correlation between OSA severity and TC/HDL‐C ratio in a cross‐sectional study.^12^ Previous study has found increased TC/HDL‐C ratio and lower HDL‐C in OSA patients.^19^ Besides, a large cross‐sectional study explored whether gender influenced the association between OSA and dyslipidemia. The author indicated that males with severe OSA were at higher risks of a hyper‐TC/HDL‐C ratio than those with AHI ≤ 30 after controlling confounding factors, but not females.^16^ Our study showed that there was a linear positive correlation between TC/HDL‐C and the risk of severe OSA. However, the differences between male and female could not be found in our stratified analyses.

The relationship between the severity of OSA and LDL‐C/HDL‐C ratio has been rarely studied. LDL‐C/HDL‐C ratio correlated positively with AHI (*ρ* = 0.28, *p* < 0.001) and negatively with the lowest arterial oxyhemoglobin saturation(L‐SpO2) (*ρ* = −0.30, *p* < 0.001) in a research conducted in both cross‐sectional and longitudinal study, while multivariate regression analysis showed that AHI (or L‐SpO2) was independently associated with LDL‐C/HDL‐C.^20^ Our results illuminated that LDL‐C/HDL‐C ratio correlated positively with AHI and ODI but negatively with minimum SaO_2_ and mean SaO_2_. Meanwhile, there was a linear correlation between the LDL‐C /HDL‐C ratio and the risk of severe OSA. These findings suggested that the increment in LDL‐C/HDL‐C ratio is proportional to the severity of OSA, which could partially increase the risk of cardiovascular events in patients with OSA. Non‐HDL‐C contained all the atherogenic lipoproteins, such as chylomicron, VLDL, LDL, and intermediate‐density lipoprotein (IDL). It was a recognized risk factor for CVD.^21^ However, non‐HDL in this study did not show a good predictive ability for the risk of severe OSA. AI showed the same prediction of severe OSA risk as TC/HDL‐C, and the results remained stable in the stratified analysis. The per SD increment of LCI doubled the risk of severe OSA in the adjusted models. Nevertheless, the prediction effect was not as good as TG/HDL‐C, TC/HDL‐C, and AI after LCI assessed as tertiles, which could relate to the calculation method of LCI.

The utilization of these indices for the prediction of OSA is biologically reasonable. Intermittent hypoxia (IH) seems to be a key factor linking OSA to dyslipidemia, systemic inflammation, oxidative stress, endothelial dysfunction, and the progression of atherosclerosis in both in vitro and in vivo models.^22^, ^23^ IH could enhance TG and cholesterol by promoting the production of enzymes and proteins, including sterol regulatory element‐binding protein 1 and stearoyl‐coenzyme desaturase‐1 in the liver.^24^ At the same time, sympathetic nervous activation had been considered as a consequence of hypoxia in OSA.^25^ It could elevate the synthesis of very low‐density lipoprotein (VLDL) and inhibit the decomposition of LDL‐C in liver through the stimulation of alpha‐1 receptors.^20^ In addition, previous studies have illuminated that blocking alpha‐1 receptors could increase HDL‐C and decrease serum TG,^26^ while beta‐adrenergic blockers had the opposite effects.^27^ Besides, norepinephrine and cortisol could both modulate hormone‐sensitive lipoproteins and alter HDL‐C synthesis.^28^ Thus, the enhancement of sympathetic nervous environment in OSA patients is not only related to CVDs and adverse cardiovascular outcomes, but may also play a critical role in the development of dyslipidemia.^29^ Given the factors, these possible mechanisms lead to the reduction in serum HDL‐C concentration and the increment in serum TG, TC, and LDL‐C, thereby increasing TG/HDL‐C, LDL‐C/HDL‐C, non‐HDL‐C, AI, and LCI in severe OSA. The results of our research showed that nontraditional lipid profiles were positively associated with severe OSA, which was consistent with the changes brought by the above mechanisms.

This study has several limitations. Firstly, it is a retrospective research. The mechanism of dyslipidemia in OSA could not be further explored. Secondly, there were no control group for OSA. Nonetheless, in consideration of the recognized fact of the association between OSA and dyslipidemia, the study is not intended to analyze such association. Thirdly, the influence of continuous positive airway pressure (CPAP) treatment was not evaluated on nontraditional lipid indices levels. Fourthly, there were fewer female subjects than male subjects in our study. Finally, the lipid‐lowering drugs we employed were not classified.

## 5 CONCLUSION

In conclusion, all of the nontraditional lipid indices (TG/HDL‐C ratio, TC/HDL‐C ratio, LDL‐C/HDL‐C ratio, non‐HDL‐C, AI, and LCI) were positively associated with the severity of OSA. Besides, the TG/HDL‐C, TC/HDL‐C, and AI could predict the risk of severe OSA better than other nontraditional lipid indices. The utilization of mentioned nontraditional lipid profiles, which were lower‐cost and easier to calculate in clinical practice, could help to determine the risk of cardiovascular diseases, and select suitable candidates for aggressive lipoprotein‐lowering therapy in OSA patients.

## Supporting information


Table S1–S5

## Data Availability

The data that support the findings of this study are available from the corresponding author upon reasonable request.
