# Supplementary material for: Association between nontraditional lipid profiles and the severity of obstructive sleep apnea: A retrospective study
Source: J Clin Lab Anal. 2022 May 16;37(17-18):e24499. doi: 10.1002/jcla.24499 (PMC10623523; doi:10.1002/jcla.24499)
Supplement: Supplementary file 1 — Table S1–S5 [file JCLA-37-e24499-s001.docx]

Additional file 1: Table. S1–5

S1 The association between TC/HDL-C ratio (per SD increment) and the risk of severe OSA in various subgroups

| Subgroups | N | OR (95%CI) | P value | P for interaction |
| --- | --- | --- | --- | --- |
| Sex |  |  |  | 0.928 |
| Male | 504 | 1.46(1.16- 1.83) | < 0.01** |  |
| Female | 131 | 1.42(0.89- 2.27) | 0.141 |  |
| Age (year) |  |  |  | 0.757 |
| <65 | 571 | 1.47(1.19- 1.82) | < 0.001*** |  |
| ≥65 | 64 | 1.65(0.80- 3.40) | 0.175 |  |
| BMI (kg/m^2^) |  |  |  | 0.688 |
| <24 | 61 | 1.82(0.71- 4.68) | 0.213 |  |
| ≥24 | 574 | 1.50(1.22- 1.85) | < 0.001*** |  |
| Smoking |  |  |  | 0.419 |
| No | 375 | 1.42(1.11- 1.81) | 0.005** |  |
| Yes | 260 | 1.70(1.18- 2.46) | 0.005** |  |
| Alcohol drinking |  |  |  | 0.922 |
| No | 397 | 1.50(1.17- 1.92) | 0.002** |  |
| Yes | 238 | 1.53(1.04- 2.25) | 0.032* |  |
| Hypertension |  |  |  | 0.798 |
| No | 297 | 1.46(1.08- 1.98) | 0.014* |  |
| Yes | 338 | 1.54(1.16- 2.05) | 0.003** |  |
| Diabetes mellitus |  |  |  | 0.676 |
| No | 511 | 1.53(1.22- 1.92) | < 0.001*** |  |
| Yes | 124 | 1.36(0.82- 2.25) | 0.235 |  |
| Coronary artery disease |  |  |  | 0.312 |
| No | 505 | 1.55(1.23- 1.96) | < 0.001*** |  |
| Yes | 130 | 1.18(0.75- 1.87) | 0.472 |  |
| Cerebral vascular disease |  |  |  | 0.683 |
| No | 577 | 1.48(1.19- 1.83) | < 0.001*** |  |
| Yes | 58 | 1.73(0.84- 3.56) | 0.138 |  |

*BMI* body mass index

Adjusted for: age, sex, BMI, smoking, alcohol drinking, hypertension, diabetes mellitus, coronary artery disease, cerebral vascular disease. In each case, the model is not adjusted for the stratification variable. * =P < 0.05; ** = P < 0.01; *** = P < 0.001

S2 The association between LDL-C/HDL-C ratio (per SD increment) and the risk of severe OSA in various subgroups

| Subgroups | N | OR (95%CI) | P value | P for interaction |
| --- | --- | --- | --- | --- |
| Sex |  |  |  | 0.746 |
| Male | 504 | 1.41(1.13- 1.75) | 0.002** |  |
| Female | 131 | 1.30(0.87- 1.95) | 0.202 |  |
| Age (year) |  |  |  | 0.513 |
| <65 | 571 | 1.39(1.14- 1.68) | < 0.01** |  |
| ≥65 | 64 | 1.82(0.80- 4.13) | 0.150 |  |
| BMI(kg/m^2^) |  |  |  | 0.448 |
| <24 | 61 | 2.07(0.75- 5.68) | 0.158 |  |
| ≥24 | 574 | 1.40(1.16- 1.69) | < 0.001*** |  |
| Smoking |  |  |  | 0.512 |
| No | 375 | 1.36(1.08- 1.71) | 0.008** |  |
| Yes | 260 | 1.56(1.11- 2.21) | 0.011* |  |
| Alcohol drinking |  |  |  | 0.838 |
| No | 397 | 1.41(1.12- 1.77) | 0.004** |  |
| Yes | 238 | 1.47(1.03- 2.08) | 0.032* |  |
| Hypertension |  |  |  | 0.817 |
| No | 297 | 1.47(1.12- 1.93) | 0.006** |  |
| Yes | 338 | 1.40(1.07- 1.84) | 0.015* |  |
| Diabetes mellitus |  |  |  | 0.384 |
| No | 511 | 1.48(1.19- 1.83) | < 0.001*** |  |
| Yes | 124 | 1.18(0.75- 1.85) | 0.469 |  |
| Coronary artery disease |  |  |  | 0.688 |
| No | 505 | 1.44(1.17- 1.78) | < 0.001*** |  |
| Yes | 130 | 1.30(0.81- 2.08) | 0.280 |  |
| Cerebral vascular disease |  |  |  | 0.528 |
| No | 577 | 1.41(1.15- 1.72) | < 0.001*** |  |
| Yes | 58 | 1.73(0.92- 3.26) | 0.088 |  |

*BMI* body mass index

Adjusted for: age, sex, BMI, smoking, alcohol drinking, hypertension, diabetes mellitus, coronary artery disease, cerebral vascular disease. In each case, the model is not adjusted for the stratification variable. * =P < 0.05; ** = P < 0.01; *** = P < 0.001

S3 The association between non-HDL-C (per SD increment) and the risk of severe OSA in various subgroups

| Subgroups | N | OR (95%CI) | P value | P for interaction |
| --- | --- | --- | --- | --- |
| Sex |  |  |  | 0.327 |
| Male | 504 | 1.46(1.17-1.81) | < 0.001*** |  |
| Female | 131 | 1.15(0.77- 1.73) | 0.496 |  |
| Age (year) |  |  |  | 0.541 |
| <65 | 571 | 1.39(1.14- 1.70) | 0.001 ** |  |
| ≥65 | 64 | 1.74(0.86- 3.52) | 0.123 |  |
| BMI(kg/m^2^) |  |  |  | 0.908 |
| <24 | 61 | 1.35(0.59- 3.06) | 0.479 |  |
| ≥24 | 574 | 1.41(1.16- 1.72) | < 0.001*** |  |
| Smoking |  |  |  | 0.146 |
| No | 375 | 1.29(1.02- 1.63) | 0.031 * |  |
| Yes | 260 | 1.76(1.24- 2.49) | 0.002 ** |  |
| Alcohol drinking |  |  |  | 0.990 |
| No | 397 | 1.44(1.13- 1.82) | 0.003 ** |  |
| Yes | 238 | 1.43(1.01- 2.03) | 0.045 * |  |
| Hypertension |  |  |  | 0.473 |
| No | 297 | 1.54(1.14- 2.09) | 0.005** |  |
| Yes | 338 | 1.33 (1.04- 1.72) | 0.025 * |  |
| Diabetes mellitus |  |  |  | 0.559 |
| No | 511 | 1.46(1.18- 1.81) | < 0.001*** |  |
| Yes | 124 | 1.25(0.78- 2.00) | 0.345 |  |
| Coronary artery disease |  |  |  | 0.359 |
| No | 505 | 1.47(1.19- 1.82) | < 0.001 *** |  |
| Yes | 130 | 1.17(0.76- 1.80) | 0.476 |  |
| Cerebral vascular disease |  |  |  | 0.645 |
| No | 577 | 1.41(1.15- 1.72) | < 0.001*** |  |
| Yes | 58 | 1.67(0.83-3.37) | 0.153 |  |

*BMI* body mass index

Adjusted for: age, sex, BMI, smoking, alcohol drinking, hypertension, diabetes mellitus, coronary artery disease, cerebral vascular disease. In each case, the model is not adjusted for the stratification variable.

* =P < 0.05; ** = P < 0.01; *** = P < 0.001

S4 The association between AI (per SD increment) and the risk of severe OSA in various subgroups

| Subgroups | N | OR (95%CI) | P value | P for interaction |
| --- | --- | --- | --- | --- |
| Sex |  |  |  | 0.928 |
| Male | 504 | 1.46(1.16- 1.83) | 0.001** |  |
| Female | 131 | 1.42(0.89- 2.27) | 0.141 |  |
| Age (year) |  |  |  | 0.757 |
| <65 | 571 | 1.47(1.19- 1.82) | < 0.001*** |  |
| ≥65 | 64 | 1.65(0.80- 3.40) | 0.175 |  |
| BMI(kg/m^2^) |  |  |  | 0.688 |
| <24 | 61 | 1.82(0.71- 4.68) | 0.213 |  |
| ≥24 | 574 | 1.50(1.22- 1.85) | < 0.001*** |  |
| Smoking |  |  |  | 0.419 |
| No | 375 | 1.42(1.11- 1.81) | 0.005** |  |
| Yes | 260 | 1.70(1.18- 2.46) | 0.005** |  |
| Alcohol drinking |  |  |  | 0.922 |
| No | 397 | 1.50(1.17- 1.92) | 0.002** |  |
| Yes | 238 | 1.53(1.04- 2.25) | 0.032* |  |
| Hypertension |  |  |  | 0.798 |
| No | 297 | 1.46(1.08- 1.98) | 0.014 * |  |
| Yes | 338 | 1.54(1.16- 2.05) | 0.003 ** |  |
| Diabetes mellitus |  |  |  | 0.676 |
| No | 511 | 1.53(1.22- 1.92) | < 0.001 *** |  |
| Yes | 124 | 1.36(0.82- 2.25) | 0.235 |  |
| Coronary artery disease |  |  |  | 0.312 |
| No | 505 | 1.55(1.23- 1.96) | < 0.001 *** |  |
| Yes | 130 | 1.18(0.75- 1.87) | 0.472 |  |
| Cerebral vascular disease |  |  |  | 0.683 |
| No | 577 | 1.48(1.19- 1.83) | < 0.001*** |  |
| Yes | 58 | 1.73(0.84- 3.56) | 0.138 |  |

*BMI* body mass index

Adjusted for: age, sex, BMI, smoking, alcohol drinking, hypertension, diabetes mellitus, coronary artery disease, cerebral vascular disease. In each case, the model is not adjusted for the stratification variable.

* =P < 0.05; ** = P < 0.01; *** = P < 0.001

S5 The association between LCI (per SD increment) and the risk of severe OSA in various subgroups

| Subgroups | N | OR (95%CI) | P value | P for interaction |
| --- | --- | --- | --- | --- |
| Sex |  |  |  | 0.456 |
| Male | 504 | 1.98(1.30- 3.01) | < 0.01 ** |  |
| Female | 131 | 3.09(0.98- 9.71) | 0.054 |  |
| Age (year) |  |  |  | 0.975 |
| <65 | 571 | 2.20(1.46- 3.30) | < 0.001*** |  |
| ≥65 | 64 | 2.13(0.31-14.67) | 0.443 |  |
| BMI(kg/m^2^) |  |  |  | 0.169 |
| <24 | 61 | 0.89(0.24-3.25) | 0.856 |  |
| ≥24 | 574 | 2.44(1.59- 3.74) | < 0.001 *** |  |
| Smoking |  |  |  | 0.809 |
| No | 375 | 2.17(1.35- 3.49) | 0.001** |  |
| Yes | 260 | 2.42(1.14- 5.15) | 0.021* |  |
| Alcohol drinking |  |  |  | 0.550 |
| No | 397 | 2.52(1.50- 4.25) | < 0.001*** |  |
| Yes | 238 | 1.94(0.98-3.84) | 0.058 |  |
| Hypertension |  |  |  | 0.428 |
| No | 297 | 2.80(1.44- 5.45) | 0.002 ** |  |
| Yes | 338 | 2.01(1.23- 3.27) | 0.005 ** |  |
| Diabetes mellitus |  |  |  | 0.338 |
| No | 511 | 2.47(1.56-3.92) | < 0.001 *** |  |
| Yes | 124 | 1.54(0.72- 3.29) | 0.262 |  |
| Coronary artery disease |  |  |  | 0.453 |
| No | 505 | 2.35(1.50- 3.68) | < 0.001 *** |  |
| Yes | 130 | 1.61(0.71-3.65) | 0.257 |  |
| Cerebral vascular disease |  |  |  | 0.487 |
| No | 577 | 2.41(1.55- 3.75) | < 0.001 *** |  |
| Yes | 58 | 1.58(0.53-4.69) | 0.413 |  |

*BMI* body mass index

Adjusted for: age, sex, BMI, smoking, alcohol drinking, hypertension, diabetes mellitus, coronary artery disease, cerebral vascular disease. In each case, the model is not adjusted for the stratification variable. * =P < 0.05; ** = P < 0.01; *** = P < 0.001
